# Predictors of Electroconvulsive Therapy Outcome in Major Depressive Disorder

**DOI:** 10.1093/ijnp/pyac070

**Published:** 2022-10-03

**Authors:** Liang Su, Yi Zhang, Yuping Jia, Junfeng Sun, David Mellor, Ti-Fei Yuan, Yifeng Xu

**Affiliations:** Department of Psychiatry, Shanghai Mental Health Center, Shanghai Jiao Tong University School of Medicine, Shanghai, China; Department of Psychiatry, Huashan Hospital, Fudan University School of Medicine, Shanghai, China; Shanghai Key Laboratory of Psychotic Disorders, Shanghai Mental Health Center, Shanghai Jiao Tong University School of Medicine, Shanghai, China; School of Biomedical Engineering and Med-X Research Institute, Shanghai Jiao Tong University, Shanghai, China; Department of Psychiatry, Shanghai Mental Health Center, Shanghai Jiao Tong University School of Medicine, Shanghai, China; School of Biomedical Engineering and Med-X Research Institute, Shanghai Jiao Tong University, Shanghai, China; Department of Psychiatry, Shanghai Mental Health Center, Shanghai Jiao Tong University School of Medicine, Shanghai, China; School of Psychology, Deakin University, Melbourne, Australia; Shanghai Key Laboratory of Psychotic Disorders, Shanghai Mental Health Center, Shanghai Jiao Tong University School of Medicine, Shanghai, China; Department of Psychiatry, Shanghai Mental Health Center, Shanghai Jiao Tong University School of Medicine, Shanghai, China; Shanghai Key Laboratory of Psychotic Disorders, Shanghai Mental Health Center, Shanghai Jiao Tong University School of Medicine, Shanghai, China

**Keywords:** Major depressive disorder, electroconvulsive therapy, growth mixture model, logistic regression

## Abstract

**Background:**

Electroconvulsive therapy (ECT) is an effective therapy for major depressive disorder (MDD) patients. However, few clinical predictors are available to predict the treatment outcome. This study aimed to characterize the response trajectories of MDD patients undergoing ECT treatment and to identify potential clinical and demographic predictors for clinical improvement.

**Methods:**

We performed a secondary analysis on data from a multicenter, randomized, blinded, controlled trial with 3 ECT modalities (bifrontal, bitemporal, unilateral). The sample consisted of 239 patients whose demographic and clinical characteristics were investigated as predictors of ECT outcomes.

**Results:**

The results of growth mixture modeling suggested there were 3 groups of MDD patients: a non-remit group (n = 17, 7.11%), a slow-response group (n = 182, 76.15%), and a rapid-response group (n = 40, 16.74%). Significant differences in age, education years, treatment protocol, types of medication used, Hamilton Depression Scale, Hamilton Anxiety Scale score, Mini-Mental State Examination score, and Clinical Global Impression score at baseline were observed across the groups.

**Conclusions:**

MDD patients exhibited distinct and clinically relevant response trajectories to ECT. The MDD patients with more severe depression at baseline are associated with a rapid response trajectory. In contrast, MDD patients with severe symptoms and older age are related to a less response trajectory. These clinical predictors may help guide treatment selection.

**Significance Statement**

We identified 3 subgroups of major depressive disorder patients with similar onset but different response rates. Depression severity at baseline and age showed vital clinical utility in predicting ECT response.

## INTRODUCTION

Electroconvulsive therapy (ECT) is an effective, rapid, and well-established treatment for major depressive disorder (MDD) patients and has been widely adopted in psychiatry services. It modifies neurotransmitter concentrations and improves neuroplasticity, contributing to its clinical efficacy (Milev et al., 2016). A recent meta-analysis found that for MDD patients, the response rate reaches 74.2%, and the remission rate reaches 52.3% after 1 acute course of ECT (Bahji et al., 2019).

To explore the response to ECT, Cinar et al. (2010) used latent class analysis to group 153 MDD patients who received ECT treatment into 5 classes with different trajectories: fast improvement, intermediate improvement, slow improvement, slow improvement with delayed onset, and a trajectory with no improvement. After treatment, those in the intermediate improvement, slow improvement, and slow improvement with delayed onset groups were still improving and did not achieve a plateau. Another study conducted the same data-driven analysis and identified 3 distinct response trajectories during a 6-week follow-up ECT treatment of 120 MDD patients (Rhebergen et al., 2015). These were rapid remission, moderate response, and non-remitting. Within this sample, elderly MDD patients were more likely to obtain a favorable outcome. However, using latent class analysis, these studies did not consider population heterogeneity within subgroups. Other studies have reported several predictors of response to ECT, including age, depressive severity, and medication failure times (Yao et al., 2019); this approach may contribute to the increasing difficulty of identifying potential predictors of ECT outcomes and contribute to mixed results of prediction studies.

Growth mixture modeling (GMM) shows a similar profile to latent class analysis but also considers heterogeneity within subgroups (Reinecke and Seddig, 2011). In this paper, we used GMM and logistic regression analysis to interrogate the data from a randomized clinical trial (Su et al., 2019) to identify potential subgroups of MDD patients who underwent ECT treatment and their potential different response trajectories. We also explored demographic and clinical variables that may predict the optimal ECT outcome based on GMM results.

## MATERIALS AND METHODS

### Participants

This secondary analysis used data (116 participants) from a multicenter ECT study (Su et al., 2019) and combined unpublished data using the same ECT modalities (123 participants, 25 bifrontal, and 98 bitemporal). The randomized clinical trial compared 3 ECT protocols (unilateral, bifrontal, bitemporal) in MDD. All the patients were MDD patients recruited from 5 hospitals in Shanghai between January 2014 and July 2016. The study was approved by the Shanghai Mental Health Center research ethics committee. After the MDD patients are determined to receive the ECT treatment, patients and their families gave written informed consent before the screening process.

All the patients were older than 18 years. They were diagnosed with MDD according to the DSM-IV-TR diagnosis as assessed by board-certified psychiatrists. Patients who had received ECT treatment within the last 6 months or were unfit for anesthesia and ECT—or currently had or had a history of schizophrenia, substance use disorder (alcohol, nicotine, methamphetamine, etc.), bipolar disorder, or other disorders such as neurodegenerative disorder—were excluded.

### Study Procedure

After baseline assessments and screening, patients were randomly assigned to the 3 ECT protocols by STATA software (StataCorp, College Station, TX, USA). The evaluator remained blind to patient allocation. Brief-pulse (1.0-ms pulse width; current amplitude 800 mA) ECT treatment was applied 3 times per week for 4 weeks. Propofol (1.0–1.5 mg/kg) anesthesia and succinylcholine (0.5 mg/kg–1.0 mg/kg) were applied for muscle relaxation. The seizure threshold was established by dose titration in the first session. Subsequent treatments were 1.5× thresholds for bitemporal and 6× thresholds for unilateral (d’Elia placement) ECT. The stimulus charge was titrated upward as required during the treatment course. Patients’ medication information before enrolling in or during ECT treatment was collected, including antidepressants, mood stabilizers, antipsychotics, and benzodiazepines. Only the antidepressants were received during the ECT treatment. The mood stabilizers, antipsychotics, and benzodiazepines were only received before enrolling in the ECT treatment. Side effects, such as headache, muscle aches, and nausea, were recorded on the last ECT treatment session (treatment session 12 or treatment session 9). The patients who terminated the ECT treatment and did not reach remission (most of them missed >3 times) or who terminated the ECT treatment when intolerable side effects occurred were regarded as withdrawn. The remission was defined as a Hamilton Depression Scale-17 (HAMD-17) score <8.

### Measures

Demographic information, such as age, sex, total episode duration in the past, current episode duration, family history of depression, times of hospitalization and relapse, and medical information, were collected at baseline. The medications and dosage that patients received before enrolling in (mood stabilizers, antipsychotics, benzodiazepines) and during the ECT treatment were recorded (antidepressants) by psychiatrists with clinical research forms. The HAMD-17, Hamilton Anxiety Scale (HAMA), and Clinical Global Impression (CGI) were completed at baseline, week 1, week 2, week 3, and after the ECT treatment (week 4). We also measured cognitive outcomes using the Mini-Mental State Examination (MMSE) score at baseline and post treatment.

### Statistical Analysis

We used Mplus 8.0 to apply the GMM on HAMD scores to identify multiple trajectory subgroups. The unconditional model was fitted (without covariates), starting with a single-class model and successively increasing the number of classes. The best-fitting model was selected according to the following criteria: (1) lowest Akaike information criterion, Bayesian information criterion (BIC), and Sample-Size Adjusted BIC; (2) higher entropy and latent class probability value (>.90); (3) *P* value of Bootstrapped Likelihood Ratio Test (BLRT) < .05. Significant BLRT suggested that the K class fit the model better than the K-1 class. The interpretability of trajectories was also considered when determining the model’s optimal class. The starting values of the models were set to 500.

After the GMM procedure, the demographic characteristics and baseline clinical scores were analyzed according to the optimal classes. The categorical variables were examined by chi-square analysis. Due to the differences in the sample size between the 3 groups, the non-parametric Kruskal-Wallis test was applied. Then, logistic regression analysis was conducted to explore the putative predictors of the classes. To avoid the overfitting of the regression model, the number of covariates should be no more than 2. The area under the curve (AUC) of the receiver operating characteristic curve was calculated to measure the model discrimination. We applied automated forward stepwise selection with variable elimination when *P* was <.05. We also completed a secondary analysis: the categorical comparison of the HAMD remission rate and the independent *t* test of the HAMD, HAMA, and CGI scores at week 1, week 2, week 3, and the end of the ECT treatment (week 4). The Benjamini–Hochberg false discovery rate (FDR) correction was applied for multiple comparisons. All the comparisons and logistic regression were completed in R 3.6.2 (R Foundation for Statistical Computing, Vienna, Austria).

## RESULTS

### HAMD and HAMA Changes in 3 ECT Modalities

A total of 239 patients were included in the analysis. The ANCOVA with baseline HAMD as covariance was conducted to explore the differences in treatment efficacy. The HAMD showed significant differences at week 2 (F = 94.905, η2 = 0.027, *P* = .049) but not at other time points (week 1: F = 1.451, η2 = 0.270, *P* = .237; week 3: F = 2.588, η2 = 0.027, *P* = .078; week 4: F = 2.106, η2 = 0.042, *P* = .127). The HAMA showed significant differences at week 3 (F = 3.052, η2 = 0.050, *P* = .032) but not other time points (week 1: F = 0.352, η2 = 0.246, *P* = .704; week 2: F = 1.486, η2 = 0.013, *P* = .228; week 4: F = 1.606, η2 = 0.206, *P* = .033) (supplementary Figure 1). Besides, the number of participants who withdrew from ECT treatment at 4 time points between 3 groups did not show significant differences between the 3 groups at each time point (supplementary Table 2). Therefore, the efficacy of the 3 ECT modalities did not show differences.

### Response Trajectories

**Figure 1.**
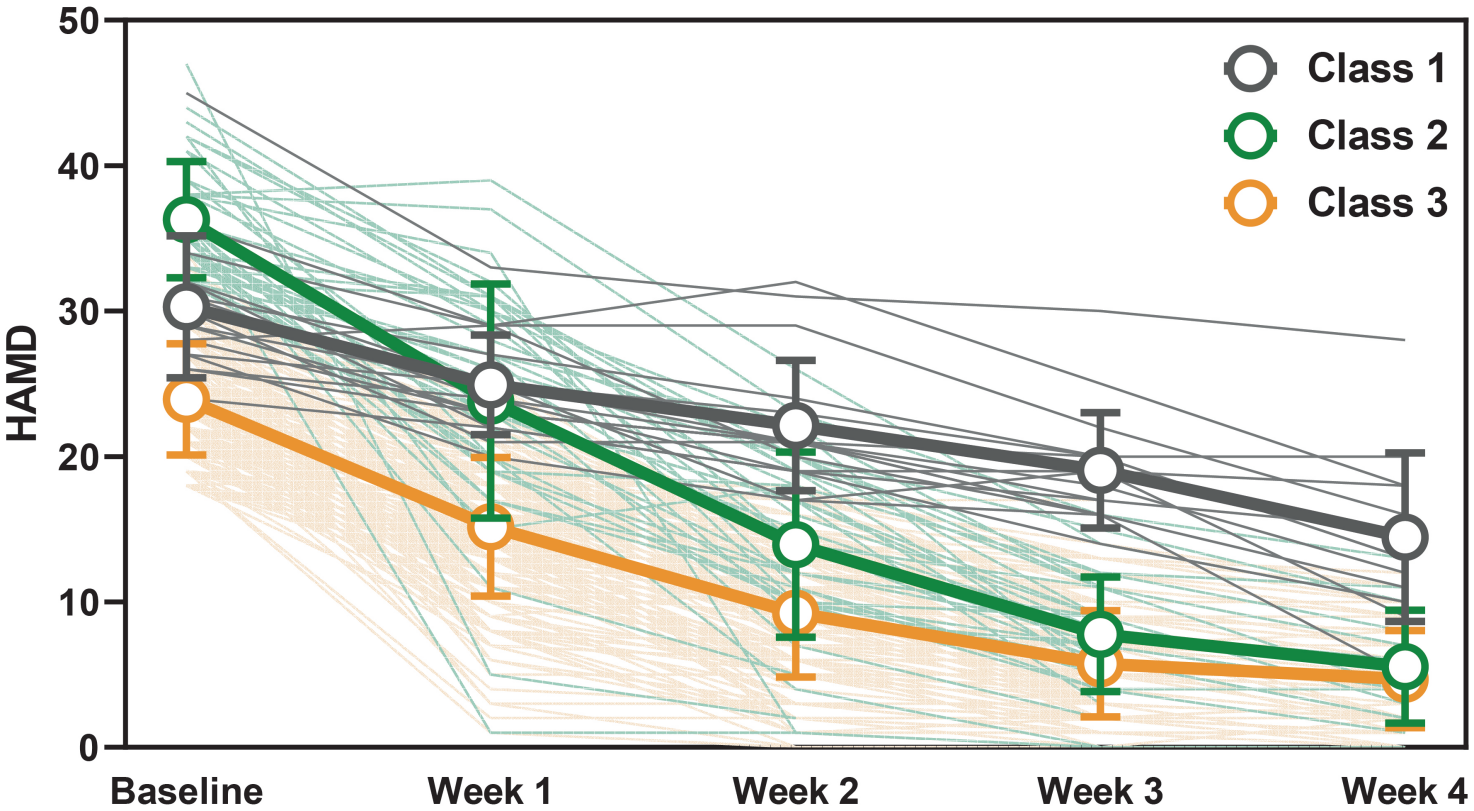
Trajectories of major depressive disorder (MDD) patients receiving electroconvulsive therapy (ECT) treatment identified by growth mixture modeling (GMM). The orange line represents the slow-response group; the green line represents the rapid-response group; and the grey line represents the non-remit group.

A comparison of models with 1, 2, 3, 4, and 5 classes suggested that the 3-class model best fit the data on most of the model selection criteria (see Table 1). The 2-class, 3-class, and 4-class models showed the significance of BLRT values, and the entropy increased as the classes increased. However, in the 4-class model, the sample size of the smallest group is smaller than 5% of the overall sample size. Further, when the number of latent classes was increased from 2 to 3, the Akaike information criterion, BIC, and Sample-Size Adjusted BIC displayed dramatic reductions, with the rate of descent becoming slower as the class number increased. Therefore, we selected the 3-class model as the best model. We labeled the 3 classes as rapid responders (n = 40, 16.74%) (unstandardized mean intercept = 36.462, unstandardized mean slope = −13.416) and slow responders (n = 182, 76.15%) (unstandardized mean intercept = 31.981, unstandardized mean slope = −6.647), and the non-responders (n = 17, 7.11%) (unstandardized mean intercept = 23.019, unstandardized mean slope = −7.623). The trajectories of the 3 classes are shown in Figure 1.

**Table 1.** Model Fit Indices of Unconditional Growth Mixture Modeling

Model	AIC	BIC	aBIC	Entropy	BLRT	Sample size
1-Class	5864.20	5909.40	5868.19	—	—	239
2-Class	5836.23	5891.85	5841.14	0.774	<0.001	49/191
3-Class	5816.12	5882.17	5821.95	0.819	<0.001	17/40/182
4-Class	5803.93	5880.41	5810.68	0.841	<0.001	10/13/66/150
5-Class	5790.88	5877.80	5798.55	0.880	0.0128	5/71/13/10/140

Abbreviations: aBIC, adjusted Bayesian information criterion; AIC, Akaike information criterion; BIC, Bayesian information criterion; BLRT, bootstrap likelihood ratio test.

### Between-Group Differences

The demographic information and baseline clinical scores of the 3 classes were compared. Group differences were found in age (χ^2^ = 13.206, FDR corrected *P* = .002), education year (χ^2^ = 6.829, FDR corrected *P* = .033), HAMD score (χ^2^ = 94.59, FDR corrected *P < *.001), HAMA score (χ^2^ = 71.961, FDR corrected *P < *.001), CGI score (χ^2^ = 16.783, FDR corrected *P < *.001), MMSE score (χ^2^ = 8.271, FDR corrected *P* = .018) (see Table 2), and treatment protocol (χ^2^ = 6.630, FDR corrected *P* = .036). The non-remit and rapid-response groups had fewer education years and were older than the slow-response group. Significant differences were observed in the medication used. The non-remit and rapid-response groups had a higher proportion of noradrenergic and selective serotonergic antidepressants use than the slow-response group. The non-remit group also had a higher proportion of typical antipsychotic use.

**Table 2.** Baseline Demographic and Clinical Characteristics in Identified Latent Trajectories in MDD Patients Receiving ECT Treatment

Characteristic	Class 1 non-remit, mean SD (n = 17)	Class 2 rapid response, mean SD (n = 40)	Class 3 slow response, mean SD (n = 182)	Statistics (FDR corrected)
**Sociodemographic**				
Age	58.15 (12.45)	55.40 (17.44)	44.63 (16.62)	χ^2^ = 13.206, *P* = .002
Education years	10.77 (4.07)	10.00 (3.45)	11.97 (3.55)	χ^2^ = 6.829, *P* = .033
Male (%)	52.94%	22.5%	38.46%	χ^2^ = 5.636, *P* = .060
BMI	21.91 (2.25)	22.39 (3.90)	22.28 (4.19)	χ^2^ = 0.902, *P* = .637
**Clinical**				
Total episode duration (m)	146.92 (148.66)	140.30 (153.00)	108.70 (113.78)	χ^2^ = 1.206, *P* = .547
Current episode duration (m)	6.12 (9.81)	3.79 (5.17)	8.18 (27.15)	χ^2^ = 0.391, *P* = .823
Times of hospitalization	2.54 (2.30)	1.43 (0.77)	1.75 (2.46)	χ^2^ = 4.578, *P* = .101
Times of relapse	2.54 (2.67)	2.23 (2.24)	2.20 (2.90)	χ^2^ = 0.780, *P* = .677
With family history	29.41%	32.5%	20.33%	χ^2^ = 3.165, *P* = .206
HAMD	30.29 (4.88)	35.75 (3.79)	23.68 (3.70)	χ^2^ = 94.59, *P < *.001
HAMA	26.54 (8.34)	32.83 (10.20)	18.18 (6.65)	χ^2^ = 71.961, *P < *.001
CGI	5.54 (0.78)	5.80 (1.19)	5.12 (0.75)	χ^2^ = 16.783, *P < *.001
MMSE	25.69 (8.22)	25.03 (4.94)	27.67 (3.09)	χ^2^ = 8.271, *P* = .018
**Treatment protocols** (No. of patients)				
Unilateral	2 (11.76%)	12 (30%)	25 (13.74%)	χ^2^ = 7.435, *P* = .024
Bifrontal	4 (23.53%)	9 (22.5%)	31 (17.03%)	χ^2^ = 0.972, *P* = .615
Bitemporal	11 (64.71%)	19 (47.5%)	126 (69.23%)	χ^2^ = 6.834, *P* = .033
**Medical** (during ECT treatment)				
**Medications** (no. of patients)				
Antidepressants	14	34	149	χ^2^ = 0.222, *P* = .895
TCAs	0	0	1	χ^2^ = 0.315, *P* = .854
SSRIs	11	24	94	χ^2^ = 1.769, *P* = .413
NaSSAs	7	21	46	χ^2^ = 12.264, *P* = .002
SNRIs	5	10	66	χ^2^ = 2.021, *P* = .364
NDRIs	1	0	5	χ^2^ = 1.861, *P* = .394
SARIs	0	2	3	χ^2^ = 2.190, *P* = .335
Agomelatine	0	1	2	χ^2^ = 0.752, *P* = .687
**Medical** (before enrolling in ECT treatment)				
**Medications** (no. of patients)				
Mood stabilizer	4	5	46	χ^2^ = 3.023, *P* = .221
Lithium carbonate	1	0	14	χ^2^ = 3.304, *P* = .192
valproate	2	3	25	χ^2^ = 1.172, *P* = .556
lamotrigine	1	2	7	χ^2^ = 0.241, *P* = .887
Typical antipsychotics (sulpiride)	2	1	2	χ^2^ = 8.674, *P* = .017
Atypical antipsychotics	7	18	103	χ^2^ = 2.900, *P* = .235
Olanzapine	3	3	30	χ^2^ = 2.164, *P* = .339
Clozapine	0	0	3	χ^2^ = 0.952, *P* = .621
Quetiapine	5	14	58	χ^2^ = 0.213, *P* = .899
Risperidone	0	1	3	χ^2^ = 0.456, *P* = .796
Aripiprazole	0	0	9	χ^2^ = 2.929, *P* = .231
BZDs	9	16	52	χ^2^ = 5.561, *P* = .062
No medication	3	3	9	χ^2^ = 4.387, *P* = .112

Abbreviations: BMI, body mass index; BZDs, benzodiazepines; CGI, Clinical Global Impression; ECT, electroconvulsive therapy; FDR, false discover rate; HAMA, Hamilton Anxiety Scale; HAMD, Hamilton Depression Scale-17; MDD, major depressive disorder; MMSE, Mini-Mental State Examination; NaSSAs: noradrenergic and selective serotonergic antidepressants; NDRIs, norepinephrine and dopamine reuptake inhibitors; non-BZDs, nonbenzodiazepine; SARIs, serotonin antagonists and reuptake inhibitors; SNRIs, serotonin and norepinephrine reuptake inhibitors; SSRIs, selective serotonin reuptake inhibitors; TCAs, tricyclic antidepressants.

For the secondary outcomes, there were significant differences between the 3 classes in the remission rate at week 2, week 3, and week 4 (week 2: χ^2^ = 31.02, FDR corrected *P < *.001; week 3: χ² = 79.47, FDR corrected *P < *.001; week 4: χ² = 23.98, FDR corrected *P < *.001) (Table 3). Moreover, the HAMD, HAMA, and CGI scores also displayed significant differences from baseline to week 4 (supplementary Table 1).

**Table 3.** HAMD Remission Rates for the 3 Latent Trajectories in MDD Patients Receiving ECT Treatment

Time points	Total sample	Non-remit	Rapid response	Slow response		
	n	n	n	n	χ²	*P*
Week 1	44 (18.49%)	0 (0%)	8 (20%)	36 (19.89%)	4.15	.125
Week 2	142 (61.47%)	0 (0%)	21 (56.76%)	121 (68.36%)	31.02	<.001
Week 3	171 (85.93%)	2 (12.5%)	32 (100%)	137 (90.73%)	79.47	<.001
Week 4	97 (93.27%)	8 (61.54%)	16 (100%)	73 (97.33%)	23.98	<.001

### Predictors of Outcome

Following the results of baseline analysis and previous research, age, gender, family history of depression, education years, current episode duration, types of medication used (tricyclic antidepressants, selective serotonin reuptake inhibitors, noradrenergic and selective serotonergic antidepressants, serotonin and norepinephrine reuptake inhibitors, norepinephrine and dopamine reuptake inhibitors, serotonin antagonists and reuptake inhibitors, agomelatine, typical antipsychotics, benzodiazepines, mood stabilizer), ECT protocol, and baseline score of HAMD, HAMA, CGI, and MMSE were entered into a multinomial logistic regression model. The latent class is the dependent variable. The results can be found in Table 4. Age (χ² = 7.650, *P* = .022) and baseline score of HAMD (χ² = 161.072, *P < *.001) were significant predictors. The medications and episode history did not enter the model (supplementary Table 3). The prediction accuracy of the model reached 92.1%. Characteristics associated with membership in the non-remit group were older age (odds ratio [OR] = 1.060, 95% confidence interval [CI] = 1.013 to 1.110, *P* = .012) and a higher baseline score of HAMD (OR = 1.448, 95% CI = 1.219 to 1.719, *P < *.001) compared with the slow-response group. The character significantly associated with the rapid-response group was a higher baseline score of HAMD (OR = 2.308, 95% CI = 1.742 to 3.059, *P < *.001), and age did not significantly associate with the membership of class 2. These indicated that the individuals with lower baseline HAMD scores and who were older responded less to the ECT treatment.

**Table 4.** Estimated Effect Sizes for the Variables Included in the Model

Classes	Variables	χ²		*P*
	Constant	208.495		<.001
	age	7.650		.022
	Baseline HAMD	161.072		<.001
		Exp(B)	95% Confidence interval	*P*
Class1	Constant			<.001
(Non-remit)	Age	1.060	[1.013 to 1.110]	.012
	Baseline HAMD	1.448	[1.219 to 1.719]	<.001
Class2	Constant			<.001
(Rapid response)	Age	1.034	[0.986 to 1.084]	.172
	Baseline HAMD	2.308	[1.742 to 3.059]	<.001
Class3	Constant			
(Slow response)	Age	1	(Reference)	
	Baseline HAMD	1	(Reference)	

**Figure 2.**
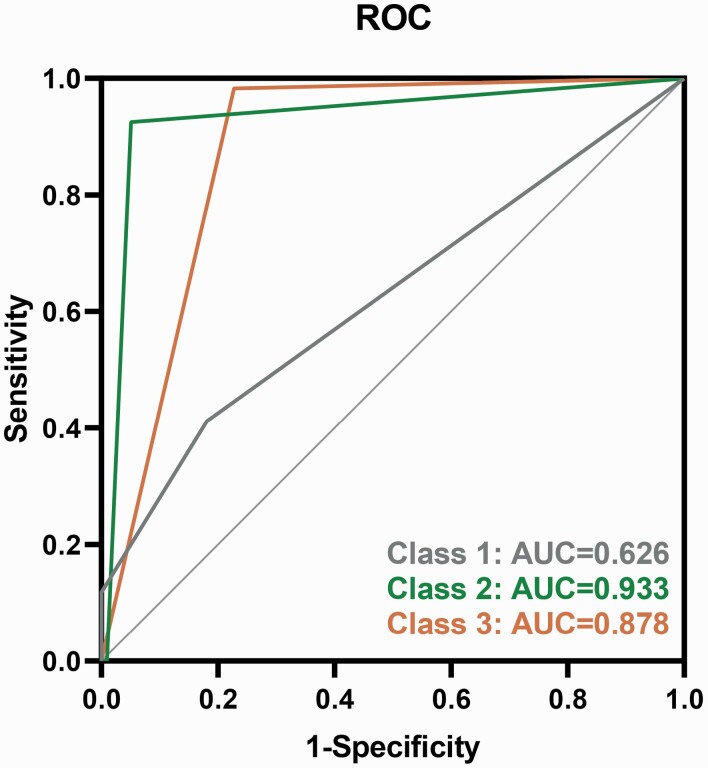
Receiver operating characteristic curve of logistic regression.

The logistic regression model indicated adequate model discrimination with an AUC of 62.6% (95% CI = 0.471% to 0.781%), which corresponded to a sensitivity of 41.2% and specificity of 81.9% (Figure 2) in the non-remit group. The AUC of the rapid-response group was 93.3% (95% CI = 0.881% to 0.984%), with a sensitivity of 92.5% and specificity of 94.9%. The AUC of the slow-response group was 87.8% (95% CI = 0.812% to 0.944%), with a sensitivity of 98.3% and specificity of 77.2%. The likelihood ratio test suggested an adequate model fit (χ² = 177.023, *P < *.001).

## DISCUSSION

The present study identified 3 distinct and clinically relevant classes of MDD patients undergoing ECT treatment. Class 1 was a non-remit group with 12.5% of patients remitted after 3 weeks, Class 2 was a rapid-response group with 100% of patients remitted after 3 weeks, and Class 3 was a slow-response group with 90.73% of patients remitted after 3 weeks. The 3 groups observed significant differences in demographic variables and clinical symptoms. The more severe depression scores were putative predictors contributing to a more rapid response to ECT treatment. In comparison, older MDD patients with severe symptoms were associated with less response to ECT treatment.

In line with previous latent class growth analysis (LCGA) analysis in ECT treatment (Uher et al., 2009; Rhebergen et al., 2015), we observed the non-remit group in the current best model. However, in the 3-class model, we observed a non-remit group with only 17 patients (7.11%), which is far lower than the percentage in previous studies (Uher et al., 2009; Rhebergen et al., 2015). This difference may be due to the different latent class methods, which can result in different classification outcomes. In addition, patients in the present study were younger (approximately 47 years old) than those in the previous ECT trajectory studies (approximately 65 years old) (Uher et al., 2009; Rhebergen et al., 2015) and showed higher remission rates (approximately 93.27% compared with approximately 50%). Besides, at week 4, only 104 patients remained in ECT treatment, indicating that the non-remit patients could withdraw during the treatment. Therefore, a high rate of withdrawal and reduced heterogeneity could have led to difficulty in differentiating between non-remit patients and remitted patients.

Based on the trajectory analysis, we identified 2 characteristics associated with the response trajectory: age and baseline HAMD score. These findings suggest that patients at an older age with severe HAMD scores are less likely to respond to the ECT treatment, but patients with more severe depressive symptoms are more likely to respond rapidly. Previous studies indicated the influence of age in the ECT outcomes (O’Connor et al., 2001; Spashett et al., 2014; Haq et al., 2015; Trevizol et al., 2019) such that age can be regarded as a reliable positive predictor of ECT treatment efficacy. Tew et al. (1999) suggested a better acute response in the older-older group (75 years and older) (Tew et al., 1999). O’Connor et al. (2001) demonstrated that MDD patients older than 46 years who were undergoing ECT treatment had a better response compared with patients younger than 46 years (O’Connor et al., 2001). Spashett et al. (2014) also revealed that older age could predict response to ECT in both psychotic and non-psychotic groups (Spashett et al., 2014). However, in the current secondary analysis study, some patients with older age did not obtain a better treatment effect but rather worse. This may be due to the different treatment protocols (unilateral, bifrontal, bitemporal). However, the protocols cannot enter the logistic model as a predictor, and there are no significant differences between the unilateral and bitemporal protocols in treatment efficacy. At the same time, our study observed that more people (30%) received unilateral protocol and fewer (47.5%) received bitemporal protocol in the rapid-response group. Van Diermen et al. (2018) indicated that the electrode placement in ECT treatment affects the influence of age (van Diermen et al., 2018). Under the right unilateral placement protocol, older age had a more substantial predictive impact than other ECT modalities (van Diermen et al., 2018).

A recent meta-analysis has demonstrated that medication failure could predict a lower response rate to ECT (Yao et al., 2019). The negative association between medication failures and treatment outcomes has also been observed in a transcranial magnetic stimulation study for the treatment of depression during pregnancy (Trevizol et al., 2019). In this study, we recorded the times of hospitalization, times of relapse, and types of medication used, none of which was associated with treatment outcomes. This phenomenon could be due to the different classification criteria, data-driven classification, and dichotomy (remission and non-remission). We identified the 3 groups (non-remit group, rapid-response group, and slow-response group), which all included remit patients. In addition, Rasmussen et al. (2007) reported that medication failure does not predict the acute remission status with ECT (Rasmussen et al., 2007).

This study has several limitations. Firstly, the present study included 3 treatment protocols, which may affect the impact of predictors, especially the unilateral protocol, which shows significant differences between the 3 groups. Though the treatment types did not contribute to the classification of the 2 groups, the underlying influence has not been entirely ruled out. Besides, we only included demographic and clinical characteristics in the model. Adding neurobiological and neurophysiological markers might improve its accuracy. For example, previous studies suggested that baseline cingulate theta cordance and fronto-temporal connectivity can predict response to ECT (DeBattista and Mueller, 2001; Leaver et al., 2018). Neurotransmitters, such as brain-derived neurotrophic factor, 5-HT_2_, and plasma noradrenaline, are also predictors (Kelly and Cooper, 1997; Okamoto et al., 2008; Yatham et al., 2018). Finally, in the current study, although board-certified psychiatrists assessed the DSM-IV-TR, HAMD, and HAMA, we did not measure the interrater reliability in the assessment of DSM-IV-TR, HAMD, and HAMA. Thus, the criteria may contain a slight bias.

In conclusion, the present study identified 3 subgroups and established a predictive model for MDD patients undergoing ECT treatment. Age and baseline HAMD score showed vital clinical utility in predicting ECT treatment response. Further studies with larger samples are required to improve the generalization of the model for clinical application.

## Supplementary Material

pyac070_suppl_Supplementary_Figure_S1

pyac070_suppl_Supplementary_Tables
