# Supplementary figures and images for: Predictors of Electroconvulsive Therapy Outcome in Major Depressive Disorder
Source: Int J Neuropsychopharmacol. 2022 Oct 3;26(1):53–60. doi: 10.1093/ijnp/pyac070 (PMC9850656; doi:10.1093/ijnp/pyac070)

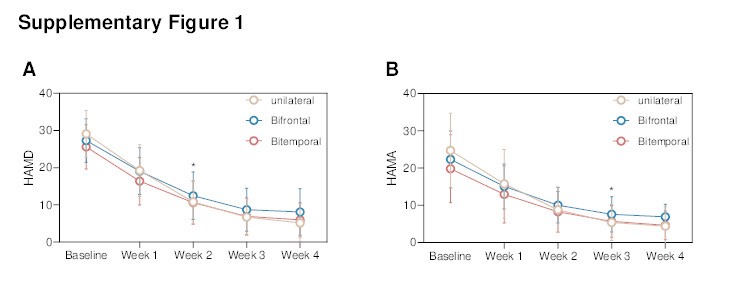

Supplement: pyac070_suppl_Supplementary_Figure_S1 [file pyac070_suppl_supplementary_figure_s1.jpeg]
