# Supplementary material for: Predictors of Electroconvulsive Therapy Outcome in Major Depressive Disorder
Source: Int J Neuropsychopharmacol. 2022 Oct 3;26(1):53–60. doi: 10.1093/ijnp/pyac070 (PMC9850656; doi:10.1093/ijnp/pyac070)
Supplement: pyac070_suppl_Supplementary_Tables [file pyac070_suppl_supplementary_tables.docx]

Supplementary figure 1. The Changes in the Hamilton Depression Scale (HAMD) scores (A) and Hamilton Anxiety Scale (HAMA) scores according to electrode placement group (B). * Represents *p*<.05.

**Supplementary Table S1. Differences in Clinical scores for the three latent trajectories at baseline, week 1, week 2, week 3, and week 4.** *P* <.005. FDR corrected.

|  |  | **Non-remit** | **Rapid response** | **Slow response** | Statistics |
| --- | --- | --- | --- | --- | --- |
| Baseline | HAMD | 30.29(4.88) | 36.30(4.00) | 23.93(3.82) | χ² = 112.266, *p*<.001 |
|  | HAMA | 25.53(8.06) | 33.15(9.95) | 18.01(6.31) | χ² =71.961, *p*<.001 |
|  | CGI | 5.47(0.80) | 5.55(1.40) | 5.09(0.78) | χ² =16.428, *p*<.001 |
| Week1 | HAMD | 24.94(3.44) | 23.83(8.07) | 15.16(4.77) | χ² =74.93, *p*<.001 |
|  | HAMA | 20.12(6.02) | 22.00(11.00) | 11.36(4.91) | χ² =58.165, *p*<.001 |
|  | CGI | 4.53(0.72) | 4.38(1.44) | 3.76(0.82) | χ² =22.913, *p*<.001 |
| Week2 | HAMD | 22.12(4.48) | 13.95(6.36) | 9.24(4.41) | χ² =60.45, *p*<.001 |
|  | HAMA | 16.00(4.61) | 12.14(7.75) | 7.23(3.89) | χ² =40.008,  *p*<.001 |
|  | CGI | 3.88(0.86) | 2.97(1.24) | 2.72(0.78) | χ² =25.205, *p*<.001 |
| Week3 | HAMD | 19.06(3.99) | 7.78(3.95) | 5.76(3.65) | χ² =49.84, *p*<.001 |
|  | HAMA | 12.75(4.99) | 7.81(6.00) | 4.77(3.09) | χ² =35.053, *p*<.001 |
|  | CGI | 3.31(0.87) | 2.31(1.40) | 2.00(0.87) | χ² =21.511, *p*<.001 |
| Week4 | HAMD | 14.46(5.80) | 5.56(3.88) | 4.68(3.36) | χ² =27.37, *p*<.001 |
|  | HAMA | 10.46(5.08) | 5.13(4.94) | 3.76(2.67) | χ² =22.629, *p*<.001 |
|  | CGI | 2.69(0.85) | 1.50(1.15) | 1.72(0.81) | χ² =12.965, *p*=.002 |

**Supplementary Table S2. Differences in ECT modalities for the three latent trajectories.**

|  | Class 1 | Class 2 | Class 3 | Total | χ^2^ | *p*-value |
| --- | --- | --- | --- | --- | --- | --- |
|  | Non-remit | Rapid response | Slow response |  |  |  |
| Bifrontal | 4 | 9 | 31 | 44 | 8.714 | 0.069 |
| Bitemporal | 11 | 19 | 126 | 156 |  |  |
| Unilateral | 2 | 12 | 25 | 39 |  |  |
| total | 17 | 40 | 182 | 239 |  |  |

**Supplementary Table S3. The completed proportion of three ECT modalities.**

|  | Total number | Unilateral  N (%) | Bifrontal  N (%) | Bitemporal  N (%) | χ^2^ | *p*-value |
| --- | --- | --- | --- | --- | --- | --- |
| Baseline | 239(100%) | 39(100%) | 44(100%) | 156(100%) | / | / |
| Week 1 | 239(100%) | 39(100%) | 44(100%) | 156(100%) | / | / |
| Week 2 | 231(96.65%) | 37(94.87%) | 43(97.73%) | 151(96.79%) | 0.549 | 0.760 |
| Week 3 | 199(83.26%) | 36(92.31%) | 34(77.27%) | 129(82.69%) | 3.459 | 0.177 |
| Week 4 | 104(43.51%) | 17(43.59%) | 13(29.55%) | 74(47.44%) | 4.469 | 0.107 |
